# Should screening for risk of gambling-related harm be undertaken in health, care and support settings? A systematic review of the international evidence

**DOI:** 10.1186/s13722-021-00243-9

**Published:** 2021-05-29

**Authors:** Lindsay Blank, Susan Baxter, Helen Buckley Woods, Elizabeth Goyder

**Affiliations:** 1 School of Health and Related Research, The University of Sheffield, Sheffield, UK; 2 Present Address: Regent Court, Regent Street Sheffield, Sheffield, S1 4DA UK

**Keywords:** Gambling, Screening, Interventions, Harm, Problem gambling, Public health

## Abstract

**Background:**

Gambling-related harm is an increasing recognised problem internationally. Recent years have seen an explosion in opportunities to gamble, both in person and online. Health and other care settings have the potential to act as screening sites to identify and support gamblers who may be at high risk of experiencing gambling-related harm. This study aimed to identify interventions to screen for risk of gambling-related harm in the general population which may be delivered in health, care and support settings.

**Methods:**

Systematic review. Searches of key databases and grey sources since 2012 were undertaken in October 2019. Electronic database searches generated a total of 5826 unique hits. Nine studies published 2013–2019, along with thirteen grey literature documents met our eligibility criteria. The criteria were setting (health, care and support settings), participants (any attendee in help, care and support settings), interventions (screening to identify risk of harm from gambling behaviours) and outcome measures (gambling behaviours, service use).

**Results:**

Three papers evaluating screening interventions delivered in general practice (repeat visits and written advice), mental health service (the use of screening tools to identify risk of harm), and substance abuse treatment (intensive outpatient treatment for substance use disorders or methadone maintenance) indicated evidence of potential effectiveness. Six papers supported the feasibility and acceptability of delivering interventions in various settings. Grey literature reports described the implementation of interventions such as training materials, and transfer of interventions developed for substance abuse populations by practitioners.

**Conclusions:**

Health, care and support services offer potentially important contexts in which to identify and offer support to people who are at risk of gambling related harm. Screening interventions appear feasible and acceptable in a range of community and healthcare settings for those at risk of gambling harm. Evaluation of effectiveness and cost-effectiveness of screening in these populations should therefore be prioritised.

## Background

Gambling is a widespread social and commercial activity, which generates substantial profits and tax revenue internationally; but can lead to addiction and harm. Gambling-related harms are the “adverse impacts from gambling on the health and wellbeing of individuals, families, communities and society” [53]. Serious harms including crime [22], intimate partner violence [12], and suicide [49] have been linked to gambling behaviours, with one notable Swedish study reporting that suicide rates in those with a diagnosed gambling disorder increased by around 15 times that of the general population [27]. The charity “Gambling With Lives” was set up in the UK by families and friends of young people who had taken their lives as a direct result of gambling [20].

Since the relaxing of gambling laws in 2005, gambling-related harm has become recognised as a serious and worsening public health problem in the UK [16] and the high social cost of gambling has been documented [5]. Gambling advertising has increased substantially [38], as have the opportunities to gamble both in the home and on mobile devices. As a result, more now needs to be done to protect and support individuals at risk of the wide range of harms related to gambling activity [28].

Gambling related harms are a relatively new concept in gambling research with a previous focus on “problem gamblers”. This is changing in recognition that the harms due to gambling are often largely determined by an individual's vulnerability to harm, due to poor mental health, social isolation or financial pressures for example, as much as the specific behaviour [53]. The term "problem gambler" has been criticised [29] as it attributes the blame for excessive gambling to "faulty" individuals [8], with blame focused on the affected individual [24]. As there is no "safe" level of gambling to be found [31] terms such as "individuals harmed by gambling" or "excessive gamblers", have been suggested in place of “problem gambling”. However, this term is still widely used in the literature.

Gambling has the potential to impact negatively on physical and psychological health, and the social functioning of the gambler and those around them—particularly for those perceived as vulnerable [55]. Various terms have been used to describe potentially harmful gambling behaviour including ‘compulsive gambling’, ‘addictive gambling’, ‘problem gambling’, and ‘pathological gambling’ [56]. These terms all refer to a pattern of excessive gambling with impaired control over gambling behaviour, significant negative consequences deriving from this impaired control, and persistence in excessive gambling despite these negative consequences [32].

Screening and brief intervention with referral to treatment where appropriate (SBIRT), is a well-established approach for tackling harmful drinking and alcohol dependence [36] as well as other substance addictions. The recognised role of health, care and support settings in facilitating this approach [6] is known to be dependent on key facilitators including providing adequate resources, training and the identification of those at risk without stereotyping [26]. However, the use of SBIRT for behavioural addictions such as gambling is less well developed.

As with substance addictions, health and other care settings have the potential to act as screening sites in order to identify and support gamblers who may be at high risk of experiencing gambling-related harm. People who already identify as “problem” gamblers are twice as likely to consult their general practitioner (GP) for mental health concerns; five times a likely to be hospital inpatients; and ten times as likely to be in receipt of psychological counselling as non-gamblers [11]. Therefore, the identification of individuals experiencing or at risk of problem gambling at an early stage has the potential to reduce harm and reduce demand on health, care and support services. Recent reviews in the field have evaluated the diagnostic accuracy of brief screening instruments designed to identify “problem gambling” in clinical settings [13, 39] e.g. Brief Biosocial Gambling Screen [4], but have not considered their applicability in wider care setting, nor their acceptability from the point of view of service providers or gamblers themselves.

In the UK there is currently no nationally recognised treatment pathway for gambling related harm. To address this lack of provision, around 15 government funded clinics are planned in the next three years as part of the National Health Service Long Term Plan [35] which will expand on services currently available. Therefore, there is a key opportunity to consider the role of screening and brief intervention as part of a developing referral pathway. This systematic review aimed to identify what is known about interventions delivered by health, care and citizen support agencies to screen for risk of gambling-related harm in the general population. It aimed to scrutinise evidence from quantitative, qualitative and discursive academic papers, together with grey literature.

## Methods

### Searches

The initial search strategy for this review combined terms for gambling with terms for screening tools. Searches were limited by date to studies published since 2012; to include a brief period of time before changes to the UK Gambling (Licensing & Advertising) Bill [19] were made. Due to time and funding constraints, searches were limited to English language publications. Citation searches on key references were also undertaken—including prior systematic reviews on similar topics. The grey literature searches were informed by the initial database searches and involved searching for a specific type of intervention ‘screening brief intervention and referral to treatment’ to find evidence of this approach used with people addicted to gambling. The full list of databases and grey literature sources which were included, along with the search terms employed, are provided in Tables 1 and 2.

**Table 1:** Databases and search terms

Databases searched	Grey sources
Medline and Medline in Process via OvidSP, Embase via OvidSP, Science Citation Index/Social Science Citation Index via Clarivate Analytics, International Bibliography of the Social Sciences via Proquest, PsycINFO via OvidSP, Social Policy and Practice via OvidSP	Social Policy and Practice via Ovid, Gordon Moody Association https://www.gordonmoody.org.uk, Be Gamble Aware https://www.begambleaware.org/, Gam Care https://www.gamcare.org.uk, Open Grey http://www.opengrey.eu/, Advanced search in Google, Web of Science Conference Proceedings (via Clarivate Analytics), Society for the Study of Addiction Conference for any references to gambling in the last five years. https://www.addiction-ssa.org/.

**Table 2:** Sample search strategy (medline)

	Search strategy
1	Gambling/
2	(gambl* or betting or lottery or lotto or lotteries or wager or electronic gambling machine*).mp
3	(problem gambl* or at risk gambl* or in transition gambl*).ti,ab
4	(disordered gambl* or excessive gambl* or 'level 2 gambl*' or destructive gambl* or compulsive gambl* or pathological gambl*).ti,ab
5	Gambling/ or "Disruptive, Impulse Control, and Conduct Disorders"/px or *Behavior, Addictive/di
6	1 or 2 or 3 or 4 or 5
7	("Rapid Screener for Problem Gambling" or RSPG or RSPG-Interview or RSPG-I or RSPG-Self-Assessment or RSPG-SA or short gambling harms scale or SGHS or "South Oaks Gambling Screen-Revised for Adolescents" or SOGS-RA).ti,ab
8	('brief self-attribution Screener for Substance and Behavioural Addictions').ti,ab
9	(SSBA or 'Brief Adolescent Gambling Screen' or BAGS or 'gambling Problem Severity Subscale' or GPSS or 'Canadian Adolescent Gambling Inventory' or CAGI or Gambling Disorder Screening Questionnaire or GDSQ or 'Early Intervention Gambling Health Test' or 'South Oaks Gambling Screen' or 'Victorian Gambling Screen' or 'Canadian Problem Gambling Index').ti,ab
10	mass screening/ or Population Surveillance/mt or *Early Diagnosis/
11	(screen* or self-screen* or self screen* or self-check* or self check* or early detection or early intervention or at risk or referral or lifestyle risk assessment).ti,ab
12	(self-help material* or counselling or harm reduction or harm minimi#ation or risk reduction or risk minimi#ation or brief motivational treatment or controlled gambl* or peer-mentor* or peer-counsellor or peer-counselor or peer counsellor or peer counselor or psycho-education* or abstinence or behavio?r management or cognitive behavio?ral therapy or brief cognitive or brief behavio?ral or trigger* or coping strategy or change talk or 'readiness to change' or decisional balance).ti,ab
13	((brief or opportunist$ or concise or short or direct or lifestyle or written or oral or verbal or personali?ed or individuali?ed) adj2 (advice or counselling or counseling or negotiation$ or guidance or discussion$ or encouragement or intervention$ or program$ or meeting$ or session$)).ti,ab
14	patient education as topic/ or health education/ or health literacy/ or directive counseling/ or counseling/ or pamphlets/
15	(patient$ education or health education or health literacy).ti,ab.
16	(patient$ adj2 (counselling or counseling or advice)).ti,ab.
17	(patient$ adj2 (leaflet$ or flyer$ or information or pamphlet$ or booklet$ or poster$)).ti,ab
18	motivational interviewing/ or harm reduction/ or risk reduction behavior/ or Behavior, Addictive/th or self-help groups/ or cognitive dissonance/ or cognitive behavioural therapy/ or exp *social support/ or therapeutic community/ or *"Disruptive, Impulse Control, and Conduct Disorders"/th
19	Primary Health Care/ or Primary prevention/ or Physicians, Family/ or general practitioners/ or physicians primary care/ or Physician-Patient Relations/ or exp general Practice/ or primary care nursing/ or Public health nursing/ or Family nursing/
20	(practice nurse$ or primary care or primary healthcare or primary health care or gp$ or general practitioner$ or family physician$ or community health).ti,ab
21	((family or general or physician$ or doctor$) adj practice$).ti,ab.
22	(GamCare or National Problem Gambling Clinic or Gordon Moody Association or Gamblers Anonymous or GamAnon or Gambling Therapy Website).ti,ab.
23	or/7–22
24	6 and 23

Database: Ovid MEDLINE(R) and Epub Ahead of Print, In-Process and Other Non-Indexed Citations, Daily and Versions(R) < 1946 to September 23, 2019 >

### Inclusion criteria

We included all studies with no limit on study design (along with grey literature sources), which considered the inclusion of screening and support for previously unidentified problem gambling in users of health, care and support services. Interventions for individuals already known to have a gambling problem, and interventions delivered by the gambling industry were excluded from the remit of this review.

### Screening

Search results were downloaded in a reference manager database (Endnote version 9, Clarivate Analytics) screened by one reviewer (with 20% checked by a second reviewer) and coded using the keyword function. Papers which were identified as potentially relevant were coded and retrieved as full paper articles. In the first instance, coding was based on title and abstract (where available) only. Where the title and abstract did not give a clear indication of whether the paper should be considered or not, an inclusive approach was taken with the full paper being considered for potential inclusion.

### Data extraction

For studies judged to be relevant, full papers were obtained and the following data were extracted and tabulated: author/year, location of study, service, setting, study approach, funding source, study design, population, findings, results/message, limitations/ concerns. Quality appraisal was undertaken for all peer-reviewed publications but not for grey literature sources. The quality appraisal tools used were specific to the study designs identified [7].

### Synthesis method

The findings were synthesised narratively and a typology of interventions and supporting evidence was developed.

## Results

**Fig 1:**
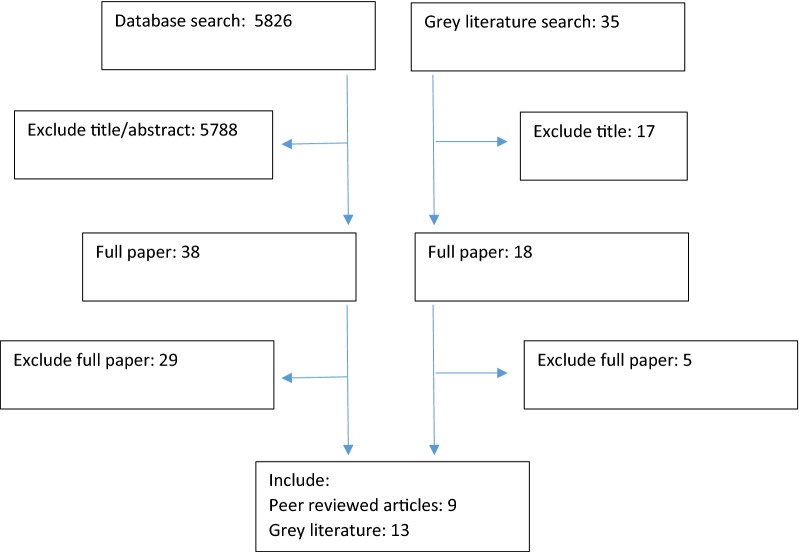
PRISMA chart

The data base searches generated a total number of 5826 unique hits (after de-duplication). Title and abstract screening identified 38 potentially relevant sources, which were obtained as full papers. Of these, 29 were excluded at the full paper stage (Fig 1). Most were excluded as they were studies of previous identified gamblers, rather than screening within the general population. This resulted in nine papers being included in the review. In addition, the grey literature search generated 35 hits of which 13 were considered to meet the review inclusion criteria. Citation searches of the included papers generated a sizable number of hits, but all these sources were subsequently found to have already been identified in the previous searches.

The searches identified two distinct sets of evidence. Firstly, we found a small set of peer reviewed research papers (n = 9) (Table 3) providing data from interventions and on practitioners’ views. Secondly, we identified “grey literature” from practice sources, typically available via websites, which described relevant interventions and often included training materials, with these delivered in a range of settings (Table 4).

**Table 3:** Included published studies

Author /year [ref] location	Setting approach	Funding source	Study design/population	Findings	Results/message	Quality appraisal/limitations
Achab 2014 [1] Switzerland	Problem gambling (PrG) screening in GP settings Attitudes and beliefs of GPs.	Le Programme Intercantonal de Lutte contre la Dependance au Jeu (PILD).	24 item online questionnaire administered to GPs. March-May 2011. N = 71 Swiss/French speaking. Age 34-71(median 53). 63.2% male.	66% response rate. 24 GPs had experience of PrG referral. 62% categorised PrG as a (very) important issue of concern. Only 7% screened for PrG in their daily practice (compared to 35% for debt screening). No relationship between screening frequency and GP interest (P = 1). Of those who had managed PrG, 52% referred to a specialist, 7% treated themselves and 32% stated they did not know what to do, and 3% did not address the issue. They reported their knowledge of PrG and specialist care networks as null (14% and 25%) or unsatisfactory ~965% and 45%). This was independent of their screening behaviour (P=0.2 and P=0.1). Most respondents reported a need for information (86%) and training (77.5%) on PrG.	GPs aware of extent and potential impact of problem gambling on their patients. However, screening isn’t systematic. Knowledge of adequate treatment or referral methods is scarce; training and information are both needed to facilitate referral.	High rates of missing data for some questions on attitudes/beliefs. Small sample size.
Dowling 2018 [14] Australia	Gambling screening in mental health services	Victorian Responsible Gambling Foundation	Comparison of brief screening instruments for problem gambling. Online survey completed in waiting rooms of mental health services June 2015-January 2016. Sample 51% M, age 38.7 (13.2).	N = 837 mental health service clinic patients Of the five screening instruments only the Brief Biosocial Gambling Screen accurately detected any level of gambling problem (low risk, moderate risk, or problem gambling). Adequate identification of problem and moderate risk gambling was achieved by NODS-CLiP and the Brief Problem Gambling Screen.	Optimum five item screening tool identified. BUT Acknowledges it is unclear what to do with patients who screen positive for gambling problems.	
Himelhoch 2015 [23] USA	Brief screening for gambling disorder in the Substance Use Treatment setting.	Not reported	Comparison of brief screens for gambling disorder Age 46.4 (10.2), African American (71%). Half met DSM-5 criteria for gambling disorder.	n=300 intensive outpatients treatment for substance use disorders or methadone maintenance programmes. Various results for gambling screening tools but suggests that commonly used brief screening tools for gambling disorder are associated with good diagnostic accuracy when used in substance use treatment settings. Client discontent in filling out screening forms (which has been a problem in previous studies) was not found to be an issue. However, 15% of those found to have a gambling disorder felt uncomfortable answering the questions. Questions were administered by researchers not linked to the patient’s treatment programme, which would not be the case in a normal context. Patients may also be concerned about their responses being shared with their clinical team.	Substance use treatment settings are suitable for screening for gambling problems. Some patient concerns over answering questions and sharing responses may need to be addressed.	Funding source not reported.
Nehlin 2016 [33] Sweden	SBIRT for gambling in primary care Staff training and interviews; patient questionnaire and support.	Public Health Agency of Sweden	Pilot study: primary care personnel trained on brief intervention (2 days); and patients who screened positive were offered a repeat visit to discuss their gambling. Those at greatest risk were also provided with written advice on seeking support. GP received financial support for participating.	N=537 screened 34 (6.3) screened positive for problem gambling. Of these, 19 of 24 at risk gamblers agreed to participate. Six completed 1 month follow up Only five of those who screened positive were female (21%). Mean age of participants was 43.7 (16.2). 5 were identified as having more serious gambling problems (more than 3 items on NODS-12 months) and were advised to seek specialist advice (written support was provided to do this). Practitioners reported that the training had been valuable. They did not find the process of administering the screening tool to be too time consuming. There was some feeling that patient had not been entirely truthful in answering the screening questions. They felt participants were more willing if asked to participate by their normal care giver (rather than being approached in the waiting room).	Staff training and support was essential. Take up from patients was low. The rate of at-risk gambling was elevated in this population suggesting that primary care is a suitable arena for gambling intervention.	Pilot study – small numbers
Roberts 2019 [44] UK	Feasibility of screening in general practice GP survey	Not reported	GP survey of views. Online survey.	N = 85 GPs. Average time as a GP = 14.67 yrs (SD 9.58, range 1–40). GPs estimated that less than 1% of patients had disclosed a gambling problem (mean 0.67), compare to 25% who discussed their smoking, and 5% drugs problems. However around 25% of GPs thought that gamblers would disclose a problem unprompted (significantly overestimating the likelihood). When asked to identify symptoms associate with problem gambling, over 75% identified financial hardship, anxiety, depression, preoccupation with gambling, stress, lying to conceal gambling and previous failed attempts to reduce gambling as indicative of problems. However, when asked to identify a care pathway for a problem gambler, only 35% of GPs were able to identify, from prior knowledge, a recognised gambling treatment provider. Other responses included “not a GP problem” or “tell them to stop” to referring to other appropriate services.	As spontaneous disclosure of problem gambling is low, GPs should be encouraged to routinely ask about gambling behaviours (as they do for substance misuse). Early detection and treatment could reduce serious mental and physical health issues associated with gambling. Overall, only 35% of GPs surveyed were able to identify, from prior knowledge, a recognised gambling treatment provider. GP knowledge of specialist referral services, and the sparsity of provision will both need to be tackled to support GPs to refer problem gambles appropriately.	Brief paper, some methodological detail missing.
Rodda 2018 [45] Australia	Views on screening for problem gambling in mental health services. Qualitative study.	Victorian Responsible Gambling Foundation	Qualitative. Interviews with clinicians and managers. January–October 2015. N = 30 clinicians and managers from 11 mental health services. 19F, 11M. Mean clinical practice 12 years (1–40). 10% had received training on how to respond to problem gambling.	Barriers to screening included a focus on immediate risk and gambling being considered as a long-term concern. Clinicians perceived problem gambling as a relatively rare condition but did acknowledge the need for brief screening. Facilitators to screening were changes to system processes, such as identification of an appropriate brief screening instrument, mandating its use as part of routine screening, as well as funded workforce development activities in the identification and management of problem gambling. Current practice was for the most part, ad hoc or at the discretion of individual clinicians. Barriers to screening were multiple and interconnected including immediate risks taking priority, seen as a rare condition and the view that tools resulted in low identification of problems Competing priorities with the requirements to screen for a range of physical and mental health conditions.	Needs to be an agreement at clinical level that brief problem gambling screening is included within a minimum dataset and routine screening practices in mental health services.	Did not seek view of other specialities.
Rogers 2013 [46] UK	Social work as a venue for screening and brief intervention of problem gamblers.	Not reported	Discussion paper	Author views: Social workers provide more support to people with problems relating to addictions that those in other helping professions. Despite this, the training of social workers and the evidence base relating to social work and addictions are sparse. As few problem gamblers actively seek treatment, efforts to improve recognition of the problem and facilitate referral to treatment would be well placed. Social workers are well placed to facilitate the management of gambling as a public health problem.	Problem gambling should be moved onto the radar of the social work profession through training programmes, research and dissemination of good practice.	Discussion paper
Sacco 2019 [48] USA	Feasibility of SBIRT screening for gambling in consumer credit counselling	National Council on Responsible Gambling	Mixed methods Routine screening for gambling in callers to a credit counselling service (Brief Bioscience Gambling Screening), Two focus groups (credit counsellors) and three key informant interviews	N = 2438. Callers were mostly female (68%), mean age 48. 52% African American, 39% White. 61% employed full time. 20% of callers to the national credit counselling agency reported gambling behaviour. Older people were most likely to be gamblers (both low and high risk) as were the full time employed and not having post-secondary level education SBIRT: screening questions were easy to incorporate, some discomfort over offering brief intervention (boundary of traditional roles), additional resources require for referral to treatment.	Credit counsellors see the benefit of screening within the service. Useful route to identify via money problems SBIRT for problem gambling is feasible in consumer credit counselling.	
Temcheff 2014 [50] Canada	Beliefs/attitudes of mental health professional on youth problem gambling	Not reported	Qualitative Online survey Child psychologists, social workers, and psychoeducators (n = 649) Female 553 (85%).	Differences between male and females as well as years of experience were assessed but no appreciable differences were found. Most able to identify characteristics of adolescent problem gamblers including preoccupation (86%), excessive time spent (79%), and increased amount of money wagered over time (81%). However, very few professionals reported knowledge of policies relating to gambling (14-18%). Viewed by most as the least serious adolescent risk behaviour (vs. drugs, alcohol and violence). BUT Strong interest in receiving continuing education in the prevention, identification, and treatment of problem gambling.	Mental health professionals felt they had a significant role to play in the prevention, identification and treatment of problem gambling. Highlighted the need for professional training.	Self-reported measures

**Table 4:** Grey literature sources

Source/funding	Setting approach	Type of information	Findings/message
Quilty [43] Gambling Research Exchange Ontario and CAMH Provincial System Support Program	Screening, Brief Intervention and Referral for Treatment in mental health services SBIRT	PowerPoint presentation and tool kit	Guided process for identifying and referring problem gamblers.
Gamble aware [18] Brief Intervention Guide	Screening and brief intervention guidance for professionals who are not specialists in the treatment of gambling	Tool kit	Guide to providing brief intervention [References are mostly substance misuse brief interventions].
Gam Care [21]	How to identify the problem, how to use a brief gambling screen and a range of current referral sources.	CPD sessions	Course for those working in frontline roles where they may encounter those affected by gambling-related harm, such as gambling industry staff, primary care workers, clinicians, advisers support workers or other healthcare professionals.
Evidence Exchange Network for mental health and addiction [15]	Mental health care	Website–links to journal article.	These findings show promise for the use of brief interventions as part of screening, and referral to treatment protocols in problem gambling.
University of Maryland [47]	Mental health/substance abuse	Webinar	Suggest screening doesn’t work well in clinical practice (illusion of addressing the issue). “…. important that clinicians have the conversation with clients about whether gambling is supporting or detracting from their MH”.
University of Maryland [54]	SBIRT Intervention for Gambling Behaviours	Clinical trial protocol	Study completion expected Oct 2019. Author contacted: study has struggled to recruit clinics and is not complete.
Centre for Addiction and Mental Health [9]	Knowledge exchange for behavioural addictions (gambling, gaming and technology use).	Website including professional training, webinars and patient self-help tool	SBIRT is a valid method for supporting problem gamblers.
Frey Society for Social Work and Research [17]	Credit counselling for gambling SBIRT	Abstract linked to full text pdf (in file)	Development of a gambling screening tool.
American Psychological Association [2]	Screening, Brief Intervention and Referral for Treatment (SBIRT) for Substance Use Disorders and Addictions	Training video	SBIRT training for professionals.
Council on compulsive gambling of Pennsylvania [10]	Using SBIRT for problem gambling in the military	Webinar (15th June 2017)	Learn how to use SBIRT for gambling in a military setting.
Northstar Problem Gambling Alliance [37]	SBIRT for problem gambling	Webinar advert (4th June 2015)	The training will cover screening for gambling problems, motivational prelude to engagement and participation in behaviour change process and, if indicated, referral to treatment.
House of gambling [25]	SBIRT for problem gambling	Video	Evidence, strategies and resources for delivering SBIRT.
Anderson [3]	Approaches to delivering SBIRT	Midwest conference	Support use of SBIRT for problem gambling.

Three papers described the use of screening and brief intervention (SBIRT) to identify people experiencing or at risk of problem gambling and related harms (intervention studies). There were a further six qualitative and discussion papers looking at the feasibility of and potential for delivering such interventions (feasibility studies). This evidence from research was further supported by grey literature examples of where screening and brief intervention approaches have been adopted. These having often been adapted from interventions developed for use in substance abuse settings by practitioners, despite the absence of a specific evidence base to support their effectiveness in gambling addiction.

The three intervention studies identified were delivered in general practice [33], a mental health support service [14], and substance abuse treatment service [1]. Feasibility and discursive reports focusing on general practice [44, 45], mental health services [50], consumer credit counselling [48] and social work [46] supported these intervention papers. Considering each setting in turn:

### General practice

The three papers relating to general practice setting were conducted in Sweden [33], Switzerland [1] and the UK [44]. All three studies included only small numbers of participants and only one delivered a SBIRT intervention, with the other two seeking GP views on the suitability and feasibility of delivering SBIRT for problem gambling. Details of the participants in each study are given in Table 3. Limitations to the quality of evidence provided include small sample sizes, high rates of missing data [1] and some lack of methodological detail [44].

The intervention study reported by Nehlin et al. [33] consisted of training (two days) primary care personnel to deliver SBIRT for problem gambling. Patients who screened positive were offered a repeat visit to discuss their gambling. Those at greatest risk were also provided with written advice on seeking support. The practices received financial support for participating. Brief quantitative data on the patients screened was collected and the intervention was further evaluated via staff interviews. Practitioners reported that the training had been valuable, and they did not find the process of administering the screening tool to be too time consuming. There were some perceptions that patients had not been entirely truthful in answering the screening questions and that participants were more willing if asked to participate by their normal caregiver (rather than being approached by research staff in the waiting room). Take up of support from patients was low.

These findings were further supported by Achab et al. [1] who evaluated the attitudes and beliefs of Swedish GPs towards problem gambling screening in GP settings. Of the 71 respondents, 62% categorised problem gambling as a (very) important issue of concern. But only 7% screened for problem gambling in their daily practice (compared to 35% for debt screening). There was no relationship between screening frequency and GP interest (P = 1). Of those who had cared for patients with problem gambling, 52% reported referring patients to a specialist, 7% had treated them themselves, 32% stated they had not known what to do, and 3% said they had not addressed the issue. They reported that their knowledge of specialist care networks was either nil or unsatisfactory. Level of knowledge was independent of their screening behaviour (P = 0.2 and P = 0.1). Most respondents reported a need for information (86%) and training (77.5%) on problem gambling.

In the study by Roberts et al. [44] over 75% of GPs identified financial hardship, anxiety, depression, preoccupation with gambling, stress, lying to conceal gambling and previous failed attempts to reduce gambling as being symptoms of gambling problems. However, when asked to identify a care pathway for a problem gambler, only 35% of GPs were able to identify a recognised gambling treatment provider.

The rate of at-risk gambling was elevated in the primary care population studied by Nehlin et al. [33] suggesting that primary care is a suitable arena for gambling intervention. However, patient reluctance to accept support [1] GP knowledge of specialist referral services, and the sparsity of provision [44] will need to be tackled to support GPs to refer problem gamblers appropriately. As spontaneous disclosure of problem gambling is low, it was suggested that GPs should be encouraged to routinely ask about gambling behaviours (as already happens for substance misuse) [44].

### Substance abuse setting

One of the three interventions evaluated was delivered in the US in a substance abuse setting and consisted of intensive outpatient treatment (n = 300) for substance use disorders or methadone maintenance [23]. The authors suggested that brief screening tools for gambling disorder may have diagnostic accuracy when used in this type of setting. Client discontent in filling out screening was not found to be an issue. However, 15% of those found to have a gambling disorder reported feeling uncomfortable answering the questions. The authors highlighted that patients may be concerned about their responses being shared with their clinical team. Therefore, while substance use treatment settings are potentially suitable locations for screening for gambling problems, there are potential issues which need to be addressed.

### Mental health services

The final intervention study was conducted in a mental health service in Australia with clinic patients (n = 837) [14]. The study compared several screening instruments used for problem gambling, of which an optimum five item tool was identified. This Brief Problem Gambling Screen asked about gambling motivations, behaviours and consequences, and was the only measure to display satisfactory sensitivity in detecting any level of gambling problem (i.e. problem, moderate‐risk and low‐risk gambling) as would be seen in mental health care settings [52]. However, the authors noted that it was unclear what to do with patients who had screened positively for gambling problems. This highlights that screening is only effective if subsequent support and intervention is available.

There were two identified feasibility studies carried out in mental health care settings [45, 50]. Firstly, Rodda et al. [45] in Australia, sought views on screening for problem gambling in mental health services via interviews with clinicians and managers (n = 30). Only 10% had received training on how to respond to problem gambling. The reported barriers to screening included a focus on immediate risks and perceiving problem gambling as a relatively rare condition. Facilitators to screening outlined were changes to system processes (such as identification of an appropriate brief screening instrument), mandating its use as part of routine screening, and funded workforce development activities in the identification and management of problem gambling.

Secondly, Temcheff et al. [50] sought the beliefs/attitudes of Canadian mental health professional on youth problem gambling (n = 649). Most were able to identify characteristics of adolescent problem gamblers including: preoccupation (86%), excessive time spent (79%), and increased amount of money wagered over time (81%). Very few professionals reported knowledge of policies relating to gambling, but there was strong interest in receiving continuing education in the prevention, identification, and treatment of problem gambling. The mental health professionals perceived that they had a significant role to play in the prevention, identification and treatment of problem gambling.

### Consumer credit counselling

Sacco et al. [48] explored the feasibility of SBIRT screening for gambling in consumer credit counselling. In this qualitative study focus groups and interviews with credit counsellors were carried out (N = 2438). The study found that 20% of callers to the national credit counselling agency reported gambling behaviour. In terms of SBIRT, counsellors felt that screening questions were easy to incorporate into their processes, although there was some discomfort over offering brief intervention (due to the boundary of traditional roles). They also perceived that additional resources would be required to facilitate referral to treatment.

### Social work

Rogers et al. [46] discussed the potential for social work in the UK to be a setting for use of SBIRT to screen problem gamblers. They argued that social workers provide more support to people with problems relating to addictions than those in other helping professions. Despite this, the training of social workers in addiction and the evidence base relating to social work and addictions were described as sparse. The authors recommended that efforts to improve recognition of problem gambling, and facilitate referral to treatment, would be well placed with gambling moved “onto the radar” of the social work profession via training programmes, research and dissemination of good practice.

### Evidence from grey literature

Thirteen grey literature sources were identified which specifically related to the delivery of SBIRT for people with suspected problem gambling. These sources provided examples of where SBIRT approaches had been transferred from substance abuse and other settings by practitioners. These examples from practice show services being provided in the absence of a substantial evidence base to support the effectiveness of this approach in problem gamblers.

The 13 sources consisted of online tool kits, material from training sessions, webinars, websites, and links to pdf reports. The grey literature searches also identified a protocol for an randomised controlled trial (RCT) of SBIRT for problem gambling [54]. The trial was due to complete in 2019. However, on contacting the trial protocol authors it was found that the research team had experienced problems in recruiting clinics to the trial and was yet to commence data collection.

This evidence indicates that in practice, SBIRT type approaches are being used in health and care settings such as mental health services [54], Evidence Exchange Network for mental health and addiction [9, 15, 47], substance misuse treatment (Gamble Aware [2, 18] primary care workers, clinicians, advisers support workers and other healthcare professionals [21], social work [17], service for military personnel [10], and general guides for using SBIRT in gambling [3, 25, 37]. However, these grey sources do not provide any information on the level of provision or the effectiveness or acceptability of the services to their clients.

## Discussion

Our review demonstrates that the evidence base for screening of potential problem gambling is in an early stage of development. However, there are key examples outside of the academic published literature of approaches such as SBIRT being used to screen and treat problem gambling in a range of settings. The effectiveness and acceptability of this approach has not been evaluated in robust studies (ideally with appropriate control groups) and so the opportunity exists to do so now. Health, care and support services are potentially vital in identifying and offering support to problem gamblers.

Screening and brief intervention appear to be feasible and acceptable in a range of community and healthcare settings, but such approaches need evaluation for effectiveness and cost-effectiveness. Other research, including a recent meta-analysis also suggests brief interventions may be effective, adding to the evidence base to support the value of earlier identification of individuals at risk from gambling-related harm [42, 42]).

Any evaluation of the effectiveness of screening interventions would also need to address service provision concerns as it would only be useful to screen people if effective support services are also available for those identified by screening as needing more support than can be offered by a brief intervention. This would be a key criterion normally for any decision about offering screening [51].

Therefore, screening must be considered in the context of developing a clear treatment pathway for problem gambling. At present there is no recognised treatment pathway and few dedicated referral services exist. The developing provision of problem gambling treatment must also be supported by training and funding for health, care and support staff to facilitate effective and timely referral.

The issues of incentivising practitioners to initiate discussion around addictive behaviours including gambling will also need to be considered if screening is shown to be effective. It has been shown that primary care consultations about alcohol consumption declined when the financial incentive to do so was removed [30]. In practice effective programmes may need to include financial incentives as well as appropriate training and support for practitioners to overcome barriers to implementation in overstretched clinical practice settings. In studies included in this review, both mental health and general practice, practitioners perceived that the treatment of gambling problems was within their role, and that it was valuable to screen for such problems. The need for staff training in gambling related harm to (include better provision and understanding of referral pathways) was also highlighted. Patient concerns around confidentiality in terms of discussing their gambling with health and care practitioners was often reported in the included studies, with this reluctance potentially presenting a key barrier to overcome in any screening programme. For example, the Productivity Commission survey in Australia reported that 60% of problem gamblers in treatment would conceal their problems in population screening questionnaires [41]. This highlights the issue that problem gamblers are almost always under-represented in prevalence surveys.

While outside the parameters of our review, there is some evidence of the effectiveness of brief interventions for problem gambling outside of health and care settings. For example, a brief intervention for problem drinking was shown to be effective in treating problem gambling in college students [34]. There is also guidance for managing gambling addiction for example for those working in the criminal justice system [40]. In addition, The Gamble Aware Brief Intervention Guidance (Gamble [18] includes guidance for "those working in social and criminal justice settings, for example social workers, employment advisers, probation officers, community workers, counsellors, GPs, nurses and psychologists" as well as those in "primary care and other healthcare settings.

### Study strengths and limitations

The broad and iterative approach to searching ensured that we were able, in the absence of a significant body of published research studies, to also include a range of evidence from practice that provided support for the feasibility and acceptability of interventions in various community settings. The dispersed and fragmented nature of this literature means it is difficult to draw firm conclusions as to the generalisability of the evidence provided by these sources. However, given the overall poor quality and low quantity of evidence available it was believed valuable to extend the review parameters to a wide range of sources.

### Implications for practice

This review has identified that there is a growing body of evidence that screening and brief intervention for people at risk of gambling harm is feasible in a range of settings and is already being delivered on a small scale and in pilot programmes. However, there is currently limited evidence for either the acceptability or effectiveness of screening and referral to specialist services in the field of gambling. The current lack of a robust evidence base suggests that further development and implementation of screening interventions should only be delivered in the context of a research study which can evaluate both effectiveness and cost-effectiveness. There is also a need for evaluation in a range of different settings to identify which are likely to be the most effective in terms of the overall aim to reduce the individual and social costs of gambling related harms.

## Conclusion

Health, care and support services are potentially vital in identifying and offering support to problem gamblers. Screening and brief intervention appear to be feasible and acceptable in a range of community and healthcare settings, but such approaches need evaluation for effectiveness, cost-effectiveness and acceptability.

## Data Availability

Not applicable (secondary data analysis).

## Abbreviations

GP: General practitioner
SBIRT: Screening and brief intervention with referral to treatment where appropriate
RCT: Randomised controlled trial
